# Rickettsia rickettsii Whole-Cell Antigens Offer Protection against Rocky Mountain Spotted Fever in the Canine Host

**DOI:** 10.1128/IAI.00628-18

**Published:** 2019-01-24

**Authors:** Andy Alhassan, Huitao Liu, Jodi McGill, Argine Cerezo, Laxmi U. M. R. Jakkula, Arathy D. S. Nair, Emma Winkley, Sally Olson, Denver Marlow, Abha Sahni, Hema P. Narra, Sanjeev Sahni, Jamie Henningson, Roman R. Ganta

**Affiliations:** a Center of Excellence for Vector-Borne Diseases, Department of Diagnostic Medicine/Pathobiology, College of Veterinary Medicine, Kansas State University (CVM-KSU), Manhattan, Kansas, USA; b Kansas State Veterinary Diagnostic Laboratory, CVM-KSU, Manhattan, Kansas, USA; c Comparative Medicine Group, CVM-KSU, Manhattan, Kansas, USA; d Department of Pathology, University of Texas Medical Branch, Galveston, Texas, USA; Washington State University

**Keywords:** RMSF, *Rickettsia*, Rocky Mountain spotted fever, tick-borne pathogens, vaccines, vector-borne diseases

## Abstract

Rocky Mountain spotted fever (RMSF) is a potentially fatal tick-borne disease in people and dogs. RMSF is reported in the United States and several countries in North, Central, and South America. The causative agent of this disease, Rickettsia rickettsii, is transmitted by several species of ticks, including Dermacentor andersoni, Rhipicephalus sanguineus, and Amblyomma americanum. RMSF clinical signs generally include fever, headache, nausea, vomiting, muscle pain, lack of appetite, and rash. If untreated, it can quickly progress into a life-threatening illness in people and dogs, with high fatality rates ranging from 30 to 80%. While RMSF has been known for over a century, recent epidemiological data suggest that the numbers of documented cases and the fatality rates remain high in people, particularly during the last two decades in parts of North America. Currently, there are no vaccines available to prevent RMSF in either dogs or people. In this study, we investigated the efficacies of two experimental vaccines, a subunit vaccine containing two recombinant outer membrane proteins as recombinant antigens (RCA) and a whole-cell inactivated antigen vaccine (WCA), in conferring protection against virulent R. rickettsii infection challenge in a newly established canine model for RMSF. Dogs vaccinated with WCA were protected from RMSF, whereas those receiving RCA developed disease similar to that of nonvaccinated R. rickettsii-infected dogs. WCA also reduced the pathogen loads to nearly undetected levels in the blood, lungs, liver, spleen, and brain and induced bacterial antigen-specific immune responses. This study provides the first evidence of the protective ability of WCA against RMSF in dogs.

## Abstract (precis)

Rocky Mountain spotted fever (RMSF) is a potentially fatal tick-borne disease in people and dogs. RMSF is reported in the United States and several countries in North, Central, and South America.

## INTRODUCTION

Rocky Mountain spotted fever (RMSF), a severe life-threatening tick-borne zoonotic disease with high fatality rates in people and dogs, is caused by the bacterium Rickettsia rickettsii (1–10). Several species of ticks harbor R. rickettsii and serve as vectors that transmit the pathogen to humans and dogs. Both dogs and people are susceptible to R. rickettsii infection. Acute and fatal cases, with mortality rates reaching between 30 and 80% in certain geographic locations of North America, are frequently documented in people, and fatal RMSF in dogs is also commonly reported (1–8). Dogs serve as the sentinel hosts for R. rickettsii due to their relatively high risk of tick exposure and susceptibility to infection (11, 12). Although Dermacentor variabilis and Dermacentor andersoni have traditionally been considered to be the primary vectors for its transmission (2, 13, 14), other hard ticks, including Amblyomma americanum and Rhipicephalus sanguineus, have also been identified as viable vectors for the RMSF pathogen (15–17). Antibiotic treatment is available for RMSF and is usually effective when implemented early during the course of disease. However, if left untreated or misdiagnosed, RMSF can cause high morbidity and mortality rates in both people and dogs (7, 18, 19). Clinical signs of RMSF in people and dogs often include high fever, lethargy, anorexia, depression, vomiting, muscle pains, edema, petechiae with ecchymoses, epistaxis, central nervous system involvement, dehydration, and weight loss (7) (https://www.cdc.gov/rmsf/symptoms/index.html). Hematological abnormalities may include anemia, thrombocytopenia, and mild leukopenia at the onset of fever, followed by leukocytosis (20–22).

Currently, no vaccines are available to prevent RMSF in either people or dogs. Vaccine development against RMSF is complicated due to the limited understanding of the protective host response and the R. rickettsii antigens involved in stimulating protective immunity. Two rickettsial outer membrane proteins (adhesion 2 [Adr2] and a segment of outer membrane protein B [OmpB-4]) have been shown to induce cell-mediated immune responses, including stimulating CD4^+^ and CD8^+^ T-cell responses in the murine host (23–26). Studies also reported that vaccination with Adr2 and OmpB-4 results in a reduced bacterial burden compared to that in unvaccinated control animals, suggesting that they may be important for the induction of a protective host response (23–26). In humans, immunization with yolk sac-grown, formalin-inactivated R. rickettsii organisms was previously shown to protect against infection challenge, and yet those studies did not progress to the development of a vaccine to prevent human or canine RMSF (27–30). Given the successful previous studies with the formalin-fixed whole-cell vaccine, we reasoned that a whole-cell inactivated vaccine will likely be protective against RMSF and may be efficiently and safely produced to prevent this debilitating disease in mammalian hosts. An obvious major advantage of a whole-cell antigen-based vaccine is the likelihood of including a broad array of potentially protective antigens to serve as immunostimulatory molecules (31–36). Whole-cell antigen-based vaccines are also known to protect vertebrate hosts (ruminants) against another endotheliotropic tick-borne rickettsial pathogen, Ehrlichia ruminantium (37–40). In this study, we assessed the potential protective abilities of two recombinant immunodominant antigens, Adr2 and OmpB-4, or the whole-cell-derived inactivated antigens of R. rickettsii for conferring protection against subsequent virulent infection challenge. As part of the vaccine studies, we also developed an infection challenge model in the canine host, which represents a natural life cycle host for R. rickettsii and is highly susceptible to severe RMSF disease. Our findings revealed that while both vaccine formulations induced antigen-specific B-cell responses, only the whole-cell-derived inactivated vaccine offered a stronger response triggering sterilizing immunity and complete protection against clinical disease and tissue pathology.

## RESULTS

### Assessment of the abilities of recombinant and whole-cell inactivated vaccines to confer protection against infection challenge with R. rickettsii.

We initiated studies to investigate the efficacies of two experimental vaccines against infection challenge of dogs with R. rickettsii. The first was a subunit vaccine composed of two immunodominant recombinant antigens (RCA) (Adr2 and OmpB-4), and the second was a heat-inactivated, whole-cell-derived vaccine preparation (WCA). Adr2 and OmpB-4 were chosen for the subunit vaccination formulation as previous studies suggested that the proteins may offer protection against RMSF because their immunization in the murine host reduces the bacterial load. The whole-cell inactivated vaccine was similarly selected because previous studies suggested that formalin-fixed R. rickettsii organisms offer protection. Recombinant Adr2 and OmpB-4 were prepared using an Escherichia coli expression system. WCA was prepared from R. rickettsii continuously cultured in Vero cells. In the first experiment, Freund’s complete adjuvant was used in the primary vaccination protocol, followed by Freund’s incomplete adjuvant for the booster vaccination. Freund’s adjuvant was selected in light of its capacity to induce strong host responses in the canine host (41–43) and because previous studies in the murine host revealed protective responses with Adr2 and OmpB-4 antigens (23–26).

Four groups of dogs (*n* = 3) were used, where one group each received WCA and RCA, one group received only adjuvants, and the last group served as unvaccinated, uninfected controls receiving only phosphate-buffered saline (PBS). Animals in the first two groups received a booster vaccination after 35 days, and similarly, the third group received a boost with only the adjuvant. Thirty-three days after the booster injections, dogs in the first three groups were intravenously (i.v.) challenged with 10^5^ R. rickettsii organisms, while the last group did not receive infection to serve as an uninfected control group. In this first study, an infectious inoculum was prepared from continuously grown, Vero cell culture-derived R. rickettsii. All 12 dogs were monitored daily for clinical signs, appetite, and behavioral changes. Dogs were also monitored weekly by complete blood count (CBC) analysis to assess for changes in their blood cell counts and profiles and for the presence of pathogen DNA.

All dogs in the first three groups developed severe inflammation at the inoculation sites, which we attributed to be the result of the use of Freund’s complete adjuvant. The inflammation subsequently progressed to persisting major puss-producing blisters. This group of dogs required detailed clinical care involving pharmacological interventions to reduce both inflammation and nonspecific wound infections. The adjuvant-associated clinical illness also prompted a delay in the booster vaccination and infection challenge by about 1 week each. The clinical signs following R. rickettsii challenge were mild in all three groups of dogs. One dog each from the RCA-vaccinated and nonvaccinated infection groups exhibited partial paralysis and mild fever, and both of these dogs were euthanized on day 11 postinfection. The remaining animals from both the RCA-vaccinated and nonvaccinated groups appeared to be normal following infection challenge, with only occasional mild fever. Dogs in the uninfected control group and the WCA-vaccinated group developed no overt clinical signs. Hematological assessment did not suggest any notable changes, with the exception of occasional rises in the neutrophil numbers and drops in hemoglobin levels and packed cell volumes (PCVs) (not shown). Nested PCR analysis to determine the presence of R. rickettsii DNA in the blood revealed only occasional DNA-positive samples in all three groups of dogs, with no notable differences between the WCA, RCA, and unvaccinated infection control groups. All noninfected control animals were negative for R. rickettsii DNA for the duration of the experiment.

**FIG 1:**
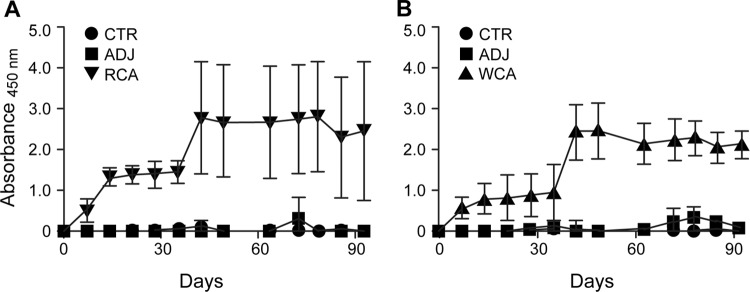
R. rickettsii-specific IgG response following vaccination and infection challenge. Antigen-specific IgG was measured in the plasma at multiple time points by an ELISA. Average absorbance values for dogs within each group were plotted against the blood sampling days. Antigens used for the ELISA included recombinant antigens (Adr2 and OmpB-4) (A) and R. rickettsii whole-cell lysate-derived antigens (B).

**FIG 2:**
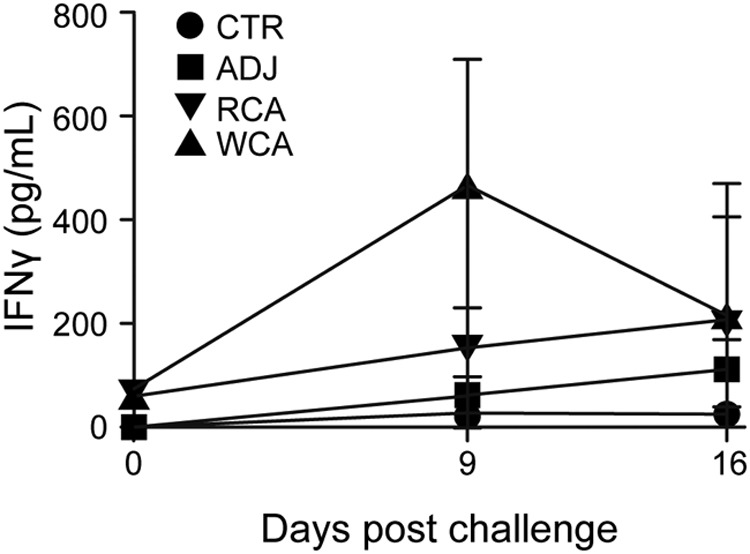
Antigen-specific IFN-γ production by PBMCs from vaccinated and challenged dogs. PBMCs were collected from all dogs on days 0, 9, and 16 after R. rickettsii challenge and isolated by density centrifugation. The cells were stimulated for 5 days with 10 μg/ml whole-cell lysate from R. rickettsii. Negative-control wells remained unstimulated. Positive-control wells were stimulated with 5 μg/ml ConA. On day 5, the cell culture supernatants were collected and analyzed by a commercial ELISA kit for the concentration of canine IFN-γ. The responses to ConA were equivalent between treatment groups, and we observed no significant differences in the responses across days (all groups combined for means ± standard errors of the means [SEM] of 4,631.1 ± 587.9 pg/ml on day 0, 4,240.7 ± 206.2 pg/ml on day 9, and 4,183.2 ± 312.6 pg/ml on day 16).

All dogs receiving WCA or RCA developed vaccine-specific IgG responses following the primary vaccination, which increased following the booster vaccination (Fig. 1). Nonvaccinated dogs had no R. rickettsii antigen-specific IgG responses. Antigen-specific gamma interferon (IFN-γ) production in peripheral blood mononuclear cells (PBMCs) was observed only for the WCA-vaccinated dogs (Fig. 2). The induction of antigen-specific IgG and IFN-γ responses following vaccination with WCA, coupled with the apparent absence of clinical signs in this group, suggested that this vaccine induced a better protective host response. However, as dogs in the nonvaccinated infection control group and in the RCA group developed milder clinical signs, we reasoned that the Vero cell culture-derived R. rickettsii infection inoculum is not sufficiently virulent to cause severe RMSF disease. Consistent with these observations, pathological assessment of various tissue samples revealed only occasional macrolesions and microlesions in infected dog tissues, independent of their vaccination status (not shown). Although the tissue lesions were consistent with RMSF, there was no apparent correlation between vaccinated and nonvaccinated dogs. Accordingly, we reasoned that the vaccine assessment studies required the following improvements: (i) development of a virulent infection model with a clear RMSF disease outcome and (ii) replacement of Freund’s complete adjuvant with a safer adjuvant, which is sufficiently immunogenic but does not induce adjuvant-associated inflammation.

### Clinical disease in dogs with egg-passaged R. rickettsii.

**FIG 3:**
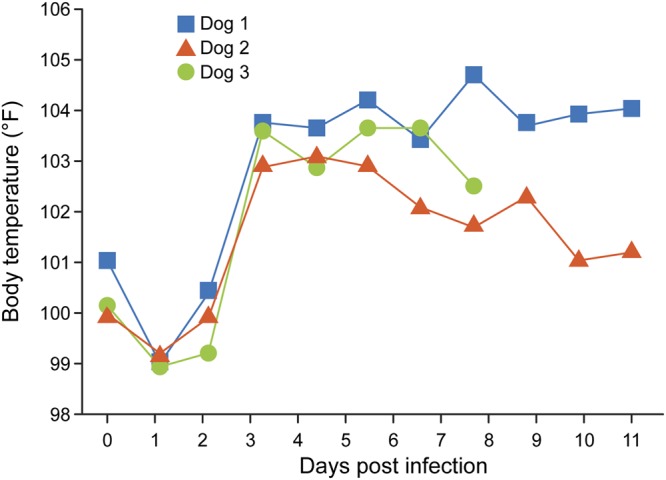
Body temperature rise in dogs receiving chicken egg embryo-raised R. rickettsii infection challenge. Rectal body temperature was monitored daily starting from the day of infection challenge (day 0) and until the day of euthanasia.

As an obligate intracellular pathogen, R. rickettsii tends to lose its virulence when continuously cultured *in vitro*, and the virulence traits may be restored if the organisms are recovered from an infected chicken embryo or passed through an animal host (44–46). To develop a model of virulent RMSF infection in dogs, we grew R. rickettsii by infecting chicken egg embryos and preparing the infectious stocks for use in the challenge inoculum. To ascertain whether egg passaging restored rickettsial virulence, three 6-month-old dogs (males) were infected with 10^5^ chicken egg embryo-derived R. rickettsii organisms via i.v. inoculation. The dogs were monitored for the development of RMSF disease, and indeed, all three dogs developed severe clinical manifestations starting from day 3 postinfection. The dogs developed persistent fevers (≥103°C) until the last day of assessment (7 to 10 days postinfection) (Fig. 3). All three dogs displayed decreased appetite and were severely depressed, as judged by their lack of interest in socializing. Furthermore, they exhibited petechial rashes on the ears, gums, buccal mucosa, and testes (see Fig. S1 in the supplemental material). The rashes and inflammation were prominent on the testes, as evidenced by the swelling of scrotal sacs and darkened scrotal skin by day 5 postinfection. The dogs also developed edema of various organs, with their facial region and feet clearly being edematous. All three dogs were euthanized (one on day 7 and the other two on day 10) because of the severity of the clinical signs. The CBC analysis, performed daily on all three animals, revealed significant drops in hemoglobin levels, red blood cell (RBC) numbers, and packed cell volumes, while elevated levels of monocytes and neutrophils were observed on the day of euthanasia (not shown). All three dogs also had persistent bacteremia, which was observed from day 2 onwards, as judged by the nested PCR analysis (not shown). Pathological assessment of various tissue samples revealed macrolesions as well as microlesions consistent with RMSF in dogs (not shown). Together, these data suggested that egg embryo-passaged R. rickettsii at a dose of 10^5^ organisms is sufficient to induce characteristic signs of RMSF in laboratory dogs.

### Assessment of the abilities of recombinant and whole-cell inactivated vaccines to confer protection against challenge with virulent R. rickettsii.

We next reevaluated the WCA and RCA vaccines for efficacy against virulent infection challenge using egg-passaged R. rickettsii as the infectious inoculum. In this experiment, we used Montanide pet gel in place of Freund’s complete adjuvant. Previous studies have documented that Montanide pet gel stimulates a strong Th1 host response without causing adjuvant-associated inflammatory disease in dogs, horses, and chickens (47–49). Likewise, Montanide adjuvant is also used for human vaccine applications, and a recent study also described its use in a human clinical trial (50–52). In addition, we used a concentration of 2.5% Montanide pet gel, which is lower than the recommended 5% concentration based on previous studies, to avoid any possibility of the development of inflammation. For this experiment, four groups of dogs were used (*n* = 6; 3 males and 3 females in each group), with the first three groups being designated the recipients of WCA, RCA, or the adjuvant (ADJ) only. The last group (*n* = 3) remained nonvaccinated and uninfected (control [CTR]) to serve as the baseline controls. All dogs in the first three groups received booster injections with the respective adjuvant-vaccine formulations or the adjuvant alone 4 weeks after the initial vaccination. Four weeks following the boosters, the first three groups of dogs were challenged by i.v. inoculation with 10^5^ egg-passaged R. rickettsii organisms. Dogs from all groups were monitored daily for injection site reactions and clinical and behavioral signs after primary and booster vaccinations and following the infection challenge.

The RCA or WCA vaccines mixed with Montanide pet gel did not induce injection site reactions or alterations in blood profiles. Following R. rickettsii infection, four dogs in the WCA group developed a mild fever only on day 2 postinfection, while the remaining two animals did not develop a febrile response. All dogs in the RCA and ADJ groups developed fever on several days postinfection beginning from day 2 (Table 1). A petechial rash typical of RMSF was observed on the ears, gums, and testes (for males) of all animals in the infection control group (ADJ) as well as in the RCA group. Only one dog from the WCA-vaccinated group developed a mild rash on the ears on day 6 after infection. Additional clinical signs included noticeable swelling on the ears, feet, snout, and testes, which was observed in several dogs in the RCA and ADJ groups. Several dogs from these two groups also became lethargic, were too weak to walk or socialize, and displayed a loss of appetite within a few days following the infection challenge. Furthermore, dogs from the RCA and ADJ groups had an average weight loss of about 7 to 8%, in contrast to the dogs in the WCA-vaccinated and uninfected control groups, which had a weight gain of 8%. Hematological assessment revealed reductions in hemoglobin levels, RBC numbers, and packed cell volumes on day 7 postinfection for dogs receiving RCA and the ADJ controls, whereas the WCA and noninfected CTR groups had normal hematological values throughout the study. Five dogs from the infection control group and four dogs from the RCA group required euthanasia by day 7 postinfection. Despite the onset of fever and development of rashes, one dog each in the RCA-vaccinated and ADJ groups improved, enabling their maintenance with supportive care until the study endpoint (day 30 postinfection). The CTR and WCA group dogs were also euthanized on day 30 after challenge.

**TABLE 1:** Body temperature assessed in dogs following infection challenge

Group	Dog (sex)	Day(s) postinfection (body temp [°F])
CTR	05 (M)	Normal
	06 (M)	Normal
	08 (M)	Normal

ADJ	04 (M)	1–7 (103, 104.6, 103.1, 104.3, 104.3, 103.8, 102.9)
	07 (M)	2 (103.1), 4–6 (103.8, 103.7, 103.4)
	09 (M)	1–2 (103.7, 103.6), 4–5 (103, 102.4), 10–11 (102.6, 102.8), 26–28 (102.6, 102.7)
	16 (F)	2–8 (103.6, 104.6, 103.6, 105.6, 104.5, 104.7, 104.6)
	17 (F)	2–8 (104, 103.7, 104, 104.6. 103.4, 103.7, 103.7)
	18 (F)	2–8 (104.1, 103, 104.9, 104.9, 104, 104.3, 102.8)

RCA	13 (M)	1–3 (104.2, 102.6), 5–7 (102.9, 103.3, 102.9)
	14 (M)	3–6 (103.3, 104.4. 102.7, 103.3)
	15 (M)	3–9 (104.7, 103.8, 103.2, 104.2, 103.4, 103.4, 102.6)
	22 (F)	2–28 (102.6–102.9)
	23 (F)	2–9 (104.3, 104.8, 104.8, 103.2)
	24 (F)	2–8 (103.1, 103.5, 103.2, 104.4, 103.9, 103.5, 103.1)

WCA	10 (M)	2 (102.9)
	11 (M)	2 (102.7)
	12 (M)	Normal
	19 (F)	2 (102.6)
	20 (F)	Normal
	21 (F)	2 (102.7)
	10 (M)	2 (102.9)

**FIG 4:**
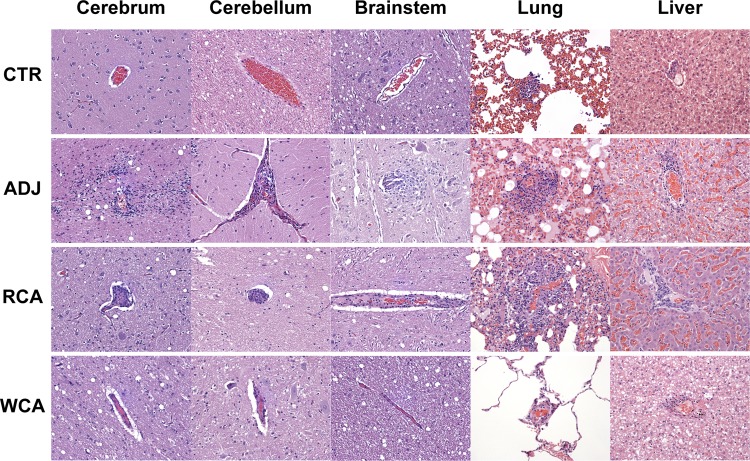
Histopathological observations in dogs impacted by vaccination. Shown are one section each of representative samples for all tissue samples (cerebrum, cerebellum, brainstem, lung, and liver) at a ×20 magnification. The histological sections were similar for CTR and WCA-vaccinated dogs, having minimal lesions attributed to a normal immune response, while the nonvaccinated ADJ and RCA-vaccinated groups had similar lesions, which are more severe than those observed in dogs belonging to the CTR and WCA groups.

**FIG 5:**
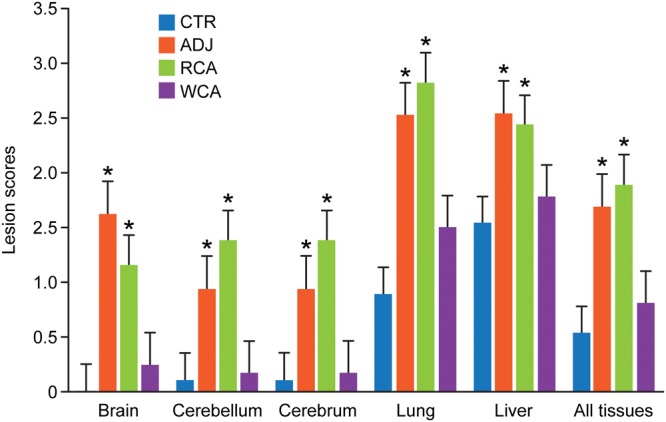
Histopathological grading scores for individual tissues and for the combined average values for all tissues. Histopathological observations of tissue samples from each dog with assigned numerical scores are presented. Bars represent mean scores ± standard deviations (SD) within each group. Asterisks above each bar refer to significant changes (*P* < 0.05) observed relative to the uninfected controls (CTR).

All main organ systems were sampled; however, we performed histopathological assessment of only cerebrum, cerebellum, brainstem, lungs, and liver as they revealed significant lesions in the infection control (ADJ) group. Similar lesions in these tissues were also noted in the RCA group animals. Dogs vaccinated with WCA and noninfected controls (CTR) had no notable lesions (Fig. 4 and 5). Pathological changes were evident in the ADJ and RCA groups for all five tissue samples histologically assessed. CTR and WCA group animals, however, did not contain significant microscopic lesions. The main lesions were characterized by a perivascular infiltrate, composed of variable numbers of lymphocytes, plasma cells, and macrophages, with occasional neutrophils (the latter being most common in lung sections). The lesions were more prominent in lungs and liver, resulting in higher lesions scores, across all groups examined. The CTR and WCA-vaccinated animals had significantly lower scores than those of the animals in the RCA and ADJ groups (*P* values of <0.05) (Fig. 5). The background inflammatory scores observed in control dogs for lung and liver tissue are attributed to a normal immune response to inhaled particles and the inherent metabolic function of the liver (53, 54). Additionally, cerebellum, cerebrum, and brainstem were also severely affected in the dogs in the ADJ and RCA groups, but the lesion scores were significantly lower for the dogs in the CTR and WCA groups.

Genomic DNA was isolated from the blood samples collected over time and from the spleen, liver, lungs, and brain tissues recovered at the study endpoint from all four groups of dogs. Samples were assessed for R. rickettsii DNA by nested PCR (Table 2). Following the infection challenge, five unvaccinated infection control dogs (ADJ) tested positive for R. rickettsii DNA in the blood starting from day 6 and remained frequently positive until the terminal day of the study. Similarly, four RCA-vaccinated dogs tested positive for R. rickettsii in the blood starting on day 6 postchallenge. In the WCA-vaccinated group, one dog tested positive on day 6, another was positive on days 6 and 8, and a third dog was positive only on day 18 after challenge (Table 2). The lungs, liver, spleen, and brain tissues collected at the termination of the study tested positive for R. rickettsii genomic DNA for 5 out of 6 dogs in the infection-only control group. Similarly, three RCA-vaccinated dogs tested positive for R. rickettsii DNA in lungs, liver, or spleen tissues. Tissue samples from all dogs from the WCA and CTR groups were negative for bacterial DNA.

**TABLE 2:** Infection status determined using blood sampled from dogs before and after infection challenge

Group	Dog	Infection status at day postchallenge												Infection status of tissue sample			
		0	2	3	6	8	9	11	13	15	18	22	26	Lung	Liver	Spleen	Brain
CTR	5		−		−	−	−	−	−	−		−	−	−	−	−	−
	6		−		−	−	−	−	−	−		−	−	−	−	−	−
	8		−		−	−	−	−	−	−		−	−	−	−	−	−

ADJ	4	−	−	−	+	+	+							−	+	+	+
	7	−	−	−	−	+	−							+	+	+	−
	9	−	−	−	+	−	−	−	−	−	−	−	−	−	−	−	−
	16	−	−	−	+	+	−							+	+	+	+
	17	−	−	−	+	−	+							−	−	+	+
	18	−	−	−	+	−	−							−	−	+	+

RCA	13	−	−	−	−	−	+	−	−	−	−			−	−	+	−
	14	−	−	−	−	−	−							−	−	−	−
	15	−	−	−	−	+	−							+	+	+	−
	22	−	−	−	−	−	−	−	−	−	−	−	−	−	−	−	−
	23	−	−	−	+	+	+							−	−	+	−
	24	−	−	−	+	+	+							−	−	−	−

WCA	10	−	−	−	−	−	−	−	−	−	−	−	−	−	−	−	−
	11	−	−	−	−	−	−	−	−	−	−	−	−	−	−	−	−
	12	−	−	−	−	−	−	−	−	−	−	−	−	−	−	−	−
	19	−	−	−	+	−	−	−	−	−	−	−		−	−	−	−
	20	−	−	−	−	−	−	−	−	−	+	−	−	−	−	−	−
	21	−	−	−	+	+	−	−	−	−	−	−		−	−	−	−

**FIG 6:**
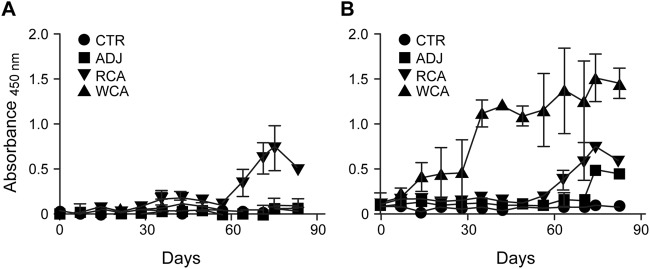
R. rickettsii-specific IgG response following vaccination and infection challenge. Antigen-specific IgG was measured in the plasma at multiple time points by an ELISA. Average absorbance values for dogs within each group were plotted against the blood sampling days. Antigens used for the ELISA included recombinant antigens (Adr2 and OmpB-4) (A) and R. rickettsii whole-cell lysate-derived antigens (B).

R. rickettsii whole-cell antigen-specific IgG responses were observed in the serum of the WCA-vaccinated dogs after both primary and booster vaccinations, with IgG levels increasing after booster vaccination and also remaining elevated after the pathogen infection challenge (Fig. 6). While RCA vaccination also induced a pathogen-specific IgG response, it was of a lower magnitude than the response in the WCA group dogs. In particular, antigen-specific IgG responses were detectable in the RCA group only after booster vaccination and following infection challenge (Fig. 6). We also performed enzyme-linked immunosorbent assays (ELISAs) with each of the two recombinant antigens individually as well as together. We observed responses for individual antigens (Adr2 and OmpB-4) very similar to those observed for the two antigens together. For simplicity, we present only the combined data. The WCA-vaccinated dogs had an IgG response to RCA that was very similar to that observed in dogs receiving the RCA vaccine (Fig. 6). The lone surviving dog in the unvaccinated infection control group also developed an R. rickettsii-specific IgG response in the serum by day 14 postinfection. As expected, uninfected dogs remained negative for the pathogen-specific antibody throughout the study.

**FIG 7:**
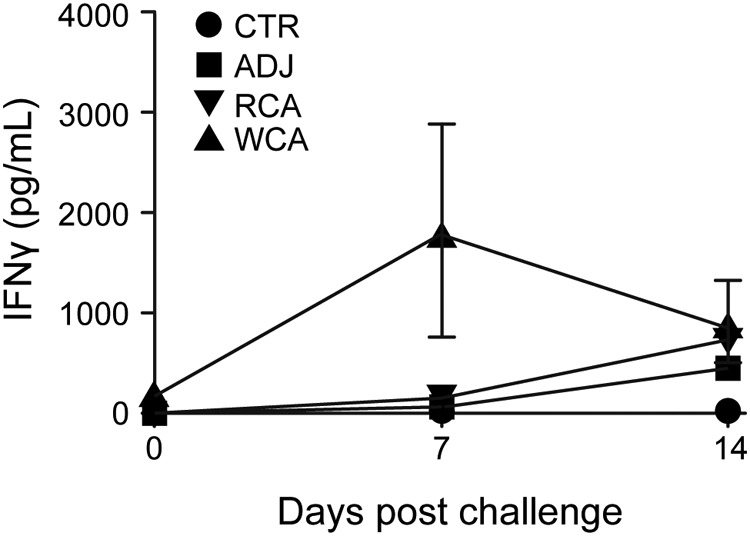
Antigen-specific IFN-γ production by PBMCs from vaccinated and challenged dogs. Peripheral blood was collected from all dogs on days 0 and 7 after R. rickettsii challenge. Peripheral blood was collected from the surviving animals on day 14 postchallenge (*n* = 1 in the unvaccinated control group, *n* = 1 in the RCA-vaccinated group, *n* = 6 in the WCA-vaccinated group, and *n* = 3 in the uninfected control group). At all time points, PBMCs were isolated by density centrifugation. The cells were stimulated for 5 days with 10 μg/ml whole-cell lysate from R. rickettsii as described in the legend of Fig. 2. Negative-control wells remained unstimulated. Positive-control wells were stimulated with 5 μg/ml ConA. On day 5, the cell culture supernatants were collected and analyzed by a commercial ELISA kit for the concentration of canine IFN-γ. The responses to ConA were equivalent between treatment groups, and we observed no significant differences in the responses across days (all groups combined for means ± SEM of 4,011.1 ± 601.0 pg/ml on day 0, 4,594.0 ± 495.6 pg/ml on day 7, and 4,343.1 ± 198.3 pg/ml on day 16).

PBMCs collected from all animals on days 0 and 7 postinfection and those that survived on day 14 after infection challenge were stimulated with whole-cell R. rickettsii antigens, and the concentration of secreted IFN-γ was measured in cell culture supernatants by an ELISA. WCA-vaccinated dogs mounted a significant IFN-γ response on day 7 after infection challenge, while no response was observed for the RCA and ADJ group dogs (Fig. 7). The RCA antigens were lethal in cell culture experiments; therefore, the antigen-specific recall responses could not be measured for either Adr2 or OmpB-4. It is unknown if the RCA-vaccinated dogs mounted a cellular immune response; thus, this may be one factor contributing to the poor level of protection in the RCA-vaccinated dogs. Antigen-specific IFN-γ secretion was absent in PBMCs isolated from all groups prior to the infection challenge (not shown).

## DISCUSSION

RMSF has been known for over a century to be one of the most lethal tick-borne diseases due to the high case fatality rates reported for R. rickettsii infections. For example, documented RMSF fatality rates in Mexico over a century range from 30 to 80% (7, 55). The disease fatality rates have remained high even in modern years (>40%) according to recent epidemiological data. Similarly, fatal RMSF cases are frequently documented in companion animals (4, 56–58). Despite its importance for human and canine health, disease diagnosis can be difficult and can result in poor prognosis if not treated early. Coupled with these challenges, measures to prevent RMSF in either dogs or people are virtually nonexistent. Therapeutic options against RMSF are limited only to tetracycline derivatives, such as doxycycline (https://www.cdc.gov/rmsf/treatment/index.html) (19, 59). Vaccine development for RMSF remains largely unexplored despite the initial efforts dating several decades back (27–29).

One of the major limitations of research on RMSF vaccines is the lack of infection models mimicking clinically relevant disease outcomes. A majority of animal studies on RMSF are carried out using the murine or guinea pig host, with a few descriptions of infection studies in dogs (3, 60, 61). Rodents do not acquire R. rickettsii infection naturally or develop RMSF as seen in humans or dogs. A second challenge to the development of RMSF vaccines has been the requirement for the use of biosafety level 3 (BSL-3) containment facilities for culturing R. rickettsii and performing certain procedures under strict biocontainment when working with infected animals. In the present study, we focused efforts on reproducing the infection model using a physiologically relevant host, the dog, and assessing the efficacies of two vaccine formulations in protecting against severe RMSF disease. In our initial experiments, continuously cultivated Vero cell culture-derived R. rickettsii organisms were used as the infection inoculum to cause RMSF in dogs. We also investigated the use of two types of vaccines: a vaccine comprised of two recombinant outer membrane protein antigens (Adr2 and OmpB-4) (RCA) or whole-organism-derived antigens (WCA) combined with Freund’s complete adjuvant. Freund’s complete adjuvant was selected because it is well known to induce a strong immune response in the canine host (41–43), and similarly, it has been reported in RCA vaccine studies in the murine host (23–26). However, the use of this adjuvant in our studies triggered a severe inflammatory disease, independent of the vaccine antigens being investigated. The adjuvant-induced sequelae required supportive care and pharmacological interventions to treat the inflammatory lesions at the vaccination sites. To our knowledge, such a phenomenon has not been well described in the literature for dogs, although Mycobacterium tuberculosis antigens present in this adjuvant have long been known to induce a strong immune response, in addition to triggering vaccine-independent inflammatory disease in various vertebrate host species (34). Thus, from this study, it became clear that this adjuvant is not well suited for canine vaccine studies, and caution must be exercised when considering this adjuvant in canine vaccine formulations.

Despite previous studies documenting that an infectious dose of 10^5^ R. rickettsii organisms is sufficient to induce severe disease in dogs (62), we did not observe the classical signs and symptoms of RMSF disease in our first study, when the inoculum was prepared from continuously cultivated Vero cell culture-derived bacteria. We reasoned that the failure to reproduce the clinical disease in the canine host might have been due to the pathogen’s loss of virulence during continuous *in vitro* propagation. To restore virulence, R. rickettsii organisms were propagated in embryonated chicken eggs (63). Indeed, i.v. inoculation of 10^5^ chicken egg embryo-derived R. rickettsii organisms was sufficient to cause classical RMSF disease within days after infection challenge in dogs. Clinical signs included persistent high fever, petechial rash, rapid discoloration of the scrotum, edema on the face and legs, rapid weight loss, reduced appetite, and the development of severe depression in all infected dogs. Pathological analysis of tissue samples collected from terminally ill animals also revealed infection-associated lesions consistent with RMSF clinical disease in the canine host.

Evidence from our studies employing this canine model of infection suggests that recombinant Adr2 and OmpB-4 are not sufficient to promote protection against R. rickettsii infection. This is in contrast to the findings reported previously from infection of the murine host, where the authors described a reduction of bacterial numbers (23, 25). Although the RCA vaccine induced an antigen-specific IgG response that was detectable following the booster vaccination, it was very low. The magnitude of the antibody response against RCA in both RCA- and WCA-vaccinated animals was significantly lower than that seen in the WCA-vaccinated dogs. Thus, it is likely that while the two recombinant antigens were protective in the murine host, they are not sufficiently immunogenic in the canine model. However, given our success in developing a canine model of R. rickettsii infection, our future studies will be aimed at further defining protective antigens in the susceptible canine host.

Furthermore, the RCA-vaccinated animals did not develop a detectable cellular immune response at any time following vaccination and challenge, suggesting that both B-cell and T-cell responses spanning a broader range of bacterial antigens are likely necessary to induce a protective host response. Importantly, this study provides the first evidence that a heat-inactivated, WCA-based R. rickettsii vaccine confers protection against fatal RMSF disease in the susceptible canine host. The WCA vaccine appeared to induce sterile immunity, as evidenced by combinatorial molecular analyses performed on genomic DNA recovered from blood sampled at various time points after infection challenge and tissue samples collected 4 weeks following the infection challenge. While the results from the present study offer the first evidence that the WCA vaccine may provide protection from intravenous R. rickettsii infection, it remains to be established if the vaccine has the capacity to induce protection from tick-transmitted R. rickettsii infection. Furthermore, additional studies are needed to define the minimum vaccine dose required and the immunological basis for vaccine-induced protection. As RMSF is an equally important disease in dogs and people, we expect that our study will lead the way forward in initiating studies to define the immune response and in identifying protective antigens needed for developing a vaccine that is valuable for both canine and human applications. Likewise, adjuvants should also be carefully formulated for applications to prevent RMSF disease in dogs and people.

In summary, this is the first study evaluating the efficacies of two types of vaccines against RMSF disease in a biologically relevant animal model, a subunit vaccine containing two immunogenic outer membrane protein antigens and a heat-inactivated, whole-organism-derived antigen vaccine. In an effort to identify the best vaccine formulations for inducing protective immunity, we also compared two adjuvants known to induce a strong and balanced immune response, Freund’s complete adjuvant and Montanide pet gel. Freund’s complete adjuvant resulted in adjuvant-associated inflammation, whereas Montanide pet gel was safe. While the RCA vaccine induced a certain degree of humoral immunity, the WCA vaccine triggered strong humoral and cellular immune responses and provided complete protection from classical canine RMSF disease.

## MATERIALS AND METHODS

### Propagation of R. rickettsii in Vero cells for preparation of stocks.

R. rickettsii (Sheila Smith strain) was grown in Vero (African green monkey kidney) cells (clone E6; ATCC CRL-1586) as previously described (64, 65). Briefly, confluent monolayers of Vero cells grown in Dulbecco’s modified Eagle’s medium (DMEM) supplemented with 2% fetal bovine serum and 2 mM l-glutamine were infected with R. rickettsii at a multiplicity of infection (MOI) of 0.1 and incubated in a 35°C incubator set at 5% CO_2_ until ∼50% of the monolayer was disrupted due to infection. The rickettsial stocks were prepared by differential centrifugation of lysates from infected cells (64) and suspended in K-36 buffer (0.1 M potassium chloride, 0.015 M sodium chloride, 0.05 M potassium phosphate buffer [pH 7.0]), and the numbers of viable R. rickettsii organisms were determined by a plaque titration assay (66, 67).

### Propagation of R. rickettsii in embryonated chicken eggs.

R. rickettsii organisms grown in Vero cells were passaged twice in specific-pathogen-free (SPF) embryonated eggs (Charles River, CT) according to established protocols (44–46). The eggs were candled using a transilluminator to mark the air sac, surface sterilized with 10% povidone-iodine, and incubated in an egg incubator with carrier rotating ability at 37.5°C with 65 to 70% humidity. The development of embryo vasculature was regularly assessed at 24-h intervals, and eggs with discordant vasculature were discarded. On day 5, a small hole was drilled into the apex of each embryonated egg, and 500 µl of a diluted R. rickettsii stock was injected using a sterile 20-gauge needle. The hole was sealed with Duco cement glue (VWR, Atlanta, GA), following which infected eggs were incubated at 34°C for several days and monitored daily for the growth of vasculature and progress of infection. All eggs exhibiting massive vasculature collapse and/or extensive hemorrhage within 72 h of infection were discarded. The vasculature from the remaining eggs was harvested on day 6 or 7 postinfection, and a small aliquot was plated on blood agar to check for contamination. The harvest aliquot from each egg was scored for the level of infection by Diff-Quick staining (Siemens, Newark, DE) and a grading system of 1 to 4 (where a score of 1 indicates a low level of infection, 2 indicates moderate infection, 3 indicates a high level of infection, and 4 indicates a very high level of infection), and R. rickettsii organisms were purified from the eggs receiving a score of 3 or 4. To ensure the complete removal of egg proteins as well as other cellular debris, the harvested material was homogenized to release bacteria and passed through a 40-µm cell strainer (Falcon, NJ). The egg harvest material containing live R. rickettsii organisms was then purified twice by differential centrifugation. Briefly, homogenized and filtered harvested egg material was centrifuged at 400 × *g* for 10 min at 4°C. The supernatant was transferred into sterile Falcon tubes, and the bacteria were pelleted by centrifugation at 15,500 × *g* for 30 min at 4°C. The final pellet was suspended in sucrose-phosphate-glutamate (SPG) buffer (0.218 M sucrose, 3.8 mM KH_2_PO_4_, 7.2 mM K_2_HPO_4_, and 4.9 mM monosodium l-glutamic acid at pH 7.0) at 1 ml per egg aliquot and stored at −80°C until use. The viability, infectivity, and stock titer were quantified by a plaque formation assay on Vero cell monolayers (66, 67). R. rickettsii stock vials were placed securely in the protective absorbent wrapping material, sealed in an approved secondary containment carrier, placed on dry ice, and shipped in consultation with the Environmental Health and Safety services at the UTMB to Kansas State University (KSU).

### Adr2 and OmpB-4 recombinant plasmid constructs and protein expression and purification.

The pET28a vector (Novagen, Madison, WI) carrying the R. rickettsii Adr2 gene and a gene fragment of OmpB-4 were prepared to prepare recombinant Adr2 and OmpB-4 proteins, respectively (25). The entire protein coding sequence of Adr2 and similarly the coding sequence of the OmpB-4 segment were amplified by PCR from R. rickettsii genomic DNA using Q5 high-fidelity DNA polymerase (New England Biolabs) with gene-specific PCR primers that encompassed the flanking NdeI site at the beginning of the forward primer end and also XhoI at the 5′ end of the reverse primer (see Table S1 in the supplemental material), respectively. PCR products were subsequently cloned into the pET28a plasmid at the above-mentioned restriction sites after digestion of both the plasmid and inserts and ligation using T4 DNA ligase. Recombinant plasmid constructs included N-terminal 6-amino-acid His tags. Recombinant Adr2 and OmpB-4 were transformed into the E. coli BL21(DE3) strain. Subsequently, the expressions of the recombinant proteins in the E. coli strain were induced by adding 1 mM isopropyl-β-d-thiogalactopyranoside to cultures growing at 30°C. The proteins were then purified using Ni-nitrilotriacetic acid (NTA)-agarose (Qiagen, Germany) according to the manufacturer’s protocols. The final concentration of purified recombinant proteins was estimated by the Bradford method using a Bio-Rad protein assay kit (Bio-Rad, Hercules, CA).

### Whole-cell antigen preparation for WCA vaccine.

Antigens for WCA were prepared by incubating the cultured organisms in a 56°C water bath for 30 min, with mixing once every 10 min. For the first vaccination experiment, WCA was prepared from Vero cell cultures. For the second experiment, the WCA vaccine was prepared from the egg-propagated organisms. The protein concentration was estimated according to the Bradford protein method (Bio-Rad, Hercules, CA).

### Vaccine formulations.

Freund’s complete adjuvant and Freund’s incomplete adjuvant (Sigma-Aldrich, St. Louis, MO) stocks at a 1:1 dilution or 2.5% Montanide pet gel (SEPPIC Inc., Fairfield, NJ) was used as the adjuvant. In the first vaccination experiment, vaccines were prepared by mixing 70 μg of whole-cell antigen (WCA) or 35 μg each of purified Adr2 and OmpB-4 antigens (RCA) in 100 μl by mixing with 100 μl of Freund’s complete or Freund’s incomplete adjuvant. In the second vaccination experiment, vaccines were similarly prepared by mixing with 70 μg of inactivated R. rickettsii whole-cell antigen or 35 μg each of purified Adr2 and OmpB-4 antigens diluted in PBS with Montanide pet gel at a final concentration of 2.5%.

### Experimental infections in dogs.

Experiments with dogs complied with *Public Health Service Policy on the Humane Care and Use of Laboratory Animals* (68) and the U.S. Department of Agriculture (USDA) Animal Welfare Act and regulations (72) and were performed with approval of the KSU Institutional Animal Care and Use Committees according to guidelines of the protocol. Purebred beagle dogs (4 to 6 months old of both sexes) were purchased from a class A USDA vendor (Covance Research Products, Denver, PA) and housed in indoor climate-controlled facilities at Kansas State University as previously described (69). Dogs were provided a commercially available dry dog food and water *ad libitum* and were also provided adequate space allowing them to freely move about for regular exercise activity. All vaccines were administered subcutaneously. All groups of dogs independent of vaccination or not, or infected or noninfected controls, were monitored daily for health, clinical, and behavioral changes and twice weekly for hematological changes by complete blood count analysis performed at the Kansas State Veterinary Diagnostic Laboratory (KSVDL) clinical pathology service section. Body weights were measured once a week. Body temperatures were measured twice a week during the vaccination phase and daily following infection challenges. Dogs were assessed for body temperatures at similar times each day, mostly between 9 and 10 a.m.

All dogs received diphenhydramine syrup (4 mg/kg body weight) about 30 min before infection challenge to avoid any possible anaphylactic shock (69). Dogs were infected with R. rickettsii (10^5^ organisms) in a 0.5-ml volume in PBS per dog via i.v. inoculation. Blood samples were collected from the cephalic vein into EDTA tubes once every 2 days from day 0, and collection continued until the study end. At the end of each experiment, all animals were euthanized in accordance with the recommendations of the Panel on Euthanasia of the American Veterinary Medical Association (AVMA), using a commercial euthanasia solution (Fatal-Plus solution at 0.22 ml/kg [86 mg/kg of pentobarbital] i.v.).

Three infection experiments were carried out: one to define RMSF disease and two in support of vaccine studies to evaluate the vaccine potential of RCA and WCA antigens. The first vaccine study included the use of Freund’s complete adjuvant as part of the primary vaccination followed by the use of Freund’s incomplete adjuvant for the booster vaccination, while the second vaccination experiment included Montanide pet gel as the adjuvant. The first vaccine study included four groups (*n* = 3), where groups 1 and 2 received WCA and RCA as the vaccine, respectively, while group 3 received only the adjuvant mixed with PBS and group 4 received only PBS. After two vaccinations administered 4 weeks apart, groups 1, 2, and 3 received infection challenge by i.v. inoculation with 1 ml of R. rickettsii culture suspensions containing 10^5^ bacteria on day 56, while group 4 did not receive infections to serve as uninfected controls. All 12 dogs were monitored daily for clinical signs of RMSF. The second infection experiment was carried out in three dogs receiving 10^5^ chicken egg-passaged R. rickettsii organisms, while the second vaccination experiment was similar to the first vaccination experiment, except that there were six dogs for each group.

### Rickettsial DNA assessed by nested PCR and IgG response assessed by an ELISA.

Whole-blood samples from dogs were collected aseptically into either 12-ml vacutainer tubes (Corning Inc., Lowell, MA) containing EDTA (Sigma-Aldrich, St. Louis, MO) or Microtainer serum separator tubes (Becton, Dickinson and Co., Franklin Lakes, NJ) for the detection of R. rickettsii DNA by PCR, and serum was used for the detection of anti-R. rickettsii antibodies. Genomic DNA was isolated from blood samples using a DNeasy blood and tissue kit (Qiagen, Valencia, CA, USA) according to the manufacturer’s protocol. DNA samples were stored at −20°C until tested by PCR for the presence of R. rickettsii DNA, and serum samples were similarly stored at −20°C until serological testing was performed. The presence of R. rickettsii DNA was assessed by a nested PCR method using Adr2 gene-specific primers (see Table S1 in the supplemental material). A negative-control reaction included a no-template PCR, and similarly, a positive-control reaction included the known R. rickettsii genomic DNA as the template. PCRs were performed in a GeneAmp 9700 instrument (Applied Biosystems, Foster City, CA). Briefly, the first round of PCR was carried out in a 25-μl reaction mixture using Platinum *Taq* DNA polymerase (Life Technologies, Carlsbad, CA, USA), and the nested PCRs were performed using 2 μl of 1:100-diluted products from the first PCR. Annealing conditions were 55°C for 30 s, extension conditions were 72°C for 60 s, and the PCRs were performed for 40 cycles. Products from the second PCR were resolved on a 1.5% agarose gel to identify predicted amplicons according to standard molecular methods (70). The presence of positive product specificity was further confirmed by sequencing of several randomly selected samples.

### Enzyme linked immunosorbent assays for R. rickettsii-specific IgG.

The ELISAs were performed using R. rickettsii inactivated whole-cell antigens or recombinant protein antigens. Serum samples collected from all dogs prior to infection and several days following vaccinations and infection challenges were assessed by ELISAs for the presence of R. rickettsii-specific IgG according to a method that we described previously for Ehrlichia chaffeensis (71). Briefly, 96-well Immulon 2HB ELISA plates (Thermo Fisher Scientific, Waltham, MA) were coated with the inactivated whole-cell R. rickettsii antigen or with recombinant Adr2 and OmpB-4 at a concentration of 20 ng/well prepared in 50 mM sodium carbonate buffer (pH 9.6). Serum samples were diluted 1:50 in PBS, added to triplicate antigen-coated wells, and incubated for 2 h at room temperature. The wells were then washed three times with PBS containing 0.05% Tween 20 (PBST) and incubated with horseradish peroxidase (HRP)-conjugated goat anti-dog total IgG (Bethyl Laboratories, Montgomery, TX) at a dilution of 1:40,000. Unbound secondary antibodies were removed by washing with PBST three times, and the specific interactions were assessed by color development using TMB (3,3′,5,5′-tetramethyl benzidine) (Calbiochem, San Diego, CA) as the substrate.

### ELISA for canine IFN-γ.

Peripheral blood mononuclear cells (PBMCs) were collected on the indicated days after RMSF challenge. Cells were isolated by density centrifugation from buffy coat fractions of peripheral blood collected into 2× acid citrate dextrose. Cells were washed and resuspended in complete RPMI composed of RPMI 1640 (Gibco, Carlsbad, CA) supplemented with 2 mM l-glutamine, 25 mM HEPES buffer, a 1% antibiotic-antimycotic solution, 50 mg/ml gentamicin sulfate, 1% nonessential amino acids, 2% essential amino acids, 1% sodium pyruvate, 50 μM 2-mercaptoethanol, and 10% (vol/vol) fetal bovine serum. Cells were cultured at 37°C with 4 × 10^5^ cells/well in 96-well plates and stimulated with 10 μg/ml whole-cell antigen. As a positive control, cells were stimulated with 5 μg/ml concanavalin A (ConA) (Sigma-Aldrich). Negative-control wells remained unstimulated. PBMC culture supernatants were collected after 5 days of stimulation, and the presence of canine IFN-γ in the supernatants was assessed by using a commercial ELISA kit (R&D Systems, Minneapolis, MN) according to the manufacturer’s instructions.

### Histopathology analysis.

Selected tissues, including cerebrum, cerebellum, brainstem, lung, liver, testicle, and epididymis, were fixed in 10% neutral buffered formalin and processed routinely with hematoxylin and eosin in 4-µm sections prepared by the KSVDL histology laboratory service. All slides were then reviewed by two pathologists (A.C. and J.H.), where they did not know sample assignments during the analysis (a blind study). A comprehensive numeric score was developed to grade the severity and distribution of inflammation in all organs examined. Inflammation within a tissue was divided into perivascular inflammation (PVI) and nonperivascular inflammation (NPVI), both of which were assigned scores ranging from 0 to 3, to evaluate subtle changes. The degree of severity of PVI was classified as follows: 0 refers to no perivascular inflammation, 1 represents the presence of 1-cell-thick perivascular cuffing, 2 refers to 2- to 3-cell-thick perivascular cuffing, and 3 signifies >3-cell-thick perivascular cuffing. Additionally, the distribution of inflammation was classified as 0 for no inflammation, 1 for single foci of inflammation, 2 for 2 to 4 foci of inflammatory infiltrates, and 3 for more than 5 inflammatory foci within a tissue examined (see Table S2 in the supplemental material). The overall predominant cell types were also assessed in each inflammatory focus for all organs assessed to distinguish perivascular from nonperivascular inflammation. A zonal distribution was noted in liver sections spanning periportal (PP) regions, central veins (CV), and other random regions (R). Similarly, we assessed for the presence of intratubular, multinucleated giant cells within the testicle and epididymis. After completion of the analysis, mean values were calculated for all tissues of each animal, and the animals’ identities were assigned to the respective experimental groups. Subsequently, organ-based inflammatory assessment scores were generated based on the average values for each animal group, and statistical analysis was performed using the ANOVA software program. Comparisons of treatment groups (ADJ, WCA, and RCA) to controls (CTR) were considered significant at a *P* value of <0.05 or different if the Bonferroni *P* value was less than 0.05.

## Supplementary Material

Supplemental file 1
