# Supplementary material for: Rickettsia rickettsii Whole-Cell Antigens Offer Protection against Rocky Mountain Spotted Fever in the Canine Host
Source: Infect Immun. 2019 Jan 24;87(2):e00628-18. doi: 10.1128/IAI.00628-18 (PMC6346123; doi:10.1128/IAI.00628-18)

Supplementary Table S1: Oligonucleotide primers used in the study.

Name	Sequences	Orientation
**For cloning *R. rickettsii* Adr2 into pET28a plasmid and testing *R. rickettssii* by PCR***		
**RG75**	5'-tgacgCATATGcttttaatagctgctacaagtgca-3'	Forward
**RG77**	5'-tgacgCTCGAGttaaaatcttataccggctgttaagttatg-3'	Reverse
**For cloning *R. rickettsii* OmpB-4 into pET28a plasmid***		
**RG78**	5'-tgacgCATATGagaggcattccattca-3'	Forward
**RG80**	5'-tgacgCTCGAGttagttctgatctacacccg-3'	Reverse
**For testing *R. rickettsii* by nest PCR**		
**Adr2-NF2**	5'-tctaatacaactgttccagttg-3'	Forward
**Adr2-NR2**	5'-ctaccatcatctatccagcta-3'	Reverse

*Capital letters with underline refer to sequences inserted for creating restriction enzyme sites. Five nucleotides were also included in the primers at the 5' end to facilitate restriction enzyme site accessibility by restriction enzymes.

Supplementary Table S2: Grading scale used in determining the histopathological scores of tissue samples assessed.

Grading perivascular inflammation

- 0 No perivascular inflammation
- 1 1 cell thick, inflammatory cells around the vessel with no involvement of the vessel wall
- 2 2-3 cell thick, perivascular inflammatory infiltrate, with no involvement of the vessel wall
- 3 More than 3 cell thick, perivascular cuffing

Grading distribution of inflammation

- 0 Absent
- 1 Focal
- 2 2-4 foci
- 3 Greater than 5

Nature of inflammation (combination of any of the following)

- L Lymphocytic
- P Plasmocytic
- H Histiocytic
- N Neutrophilic

Hemorrhage

- 0 Absent
- 1 Present

Grading distribution/severity of non-perivascular inflammatory nodules

- 0 Absent
- 1 Focal
- 2 2-4 foci
- 3 Greater than 5

Nature of inflammation (combination of any of the following)

- L Lymphocytic
- P Plasmacytic
- H Histiocytic
- N Neutrophilic

Anatomical distribution (liver)

- Cv Central vein
- Pp Periportal
- R Random

Intratubular multinuclear giant cells (testicle and epididimus)

- 0 None
- 1 Present

Supplementary Figure 1

Petechial skin rashes on a ear

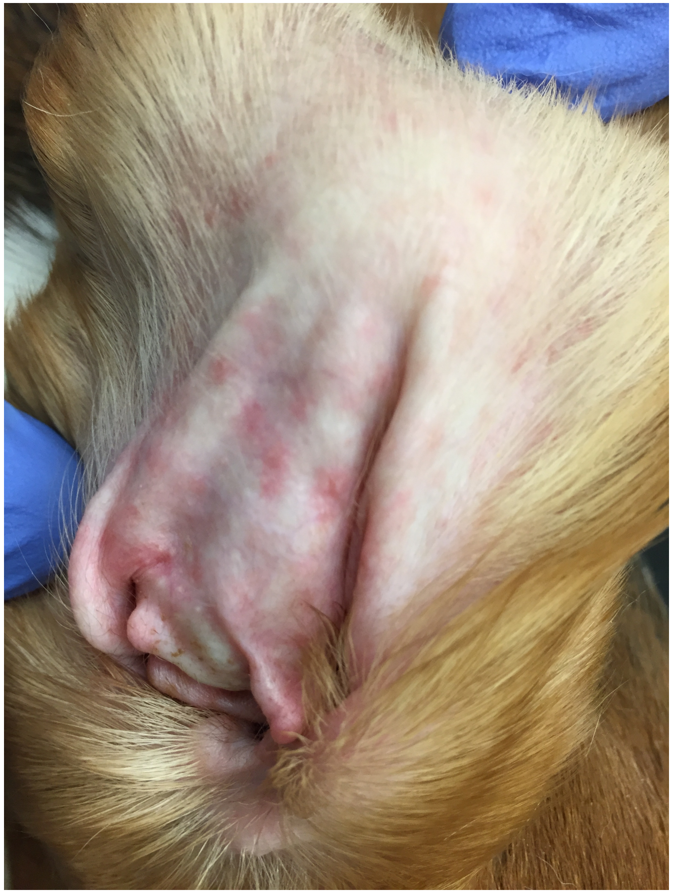

Darkened skin on a testes

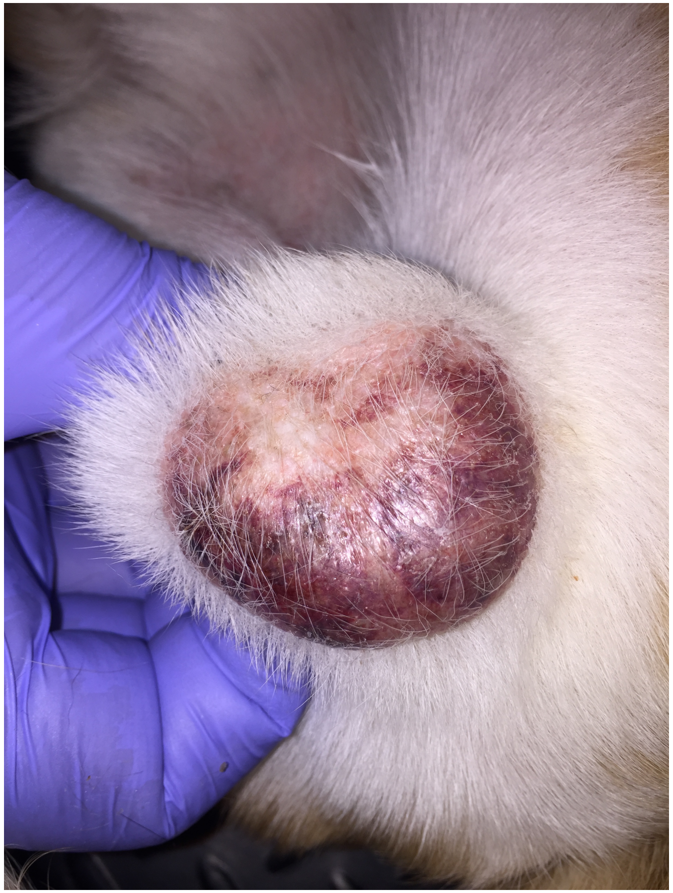

Supplement: Supplemental file 1 [file 6609fbba5065d27bc3a6f0c43a66a11c_IAI.00628-18-s0001.pdf]
